# Spontane intrakranielle Hypotension mit „brain sagging“ und reversibler frontotemporaler Demenz: Fallbericht und Übersicht über die Literatur

**DOI:** 10.1007/s00115-022-01280-8

**Published:** 2022-04-01

**Authors:** C. Hagemann, M. Christ, C. Maurer, H. Wegerer, M. Naumann, A. Bayas

**Affiliations:** 1 Klinik für Neurologie und klinische Neurophysiologie, Universitätsklinikum Augsburg, Stenglinstr. 2, 86156 Augsburg, Deutschland; 2 Klinik für Diagnostische Radiologie und Neuroradiologie, Universitätsklinikum Augsburg, Augsburg, Deutschland

## Spontane intrakranielle Hypotension

Das Leitsymptom der spontanen intrakraniellen Hypotension (SIH) ist der orthostatische Kopfschmerz. Begleitend kann es zu Übelkeit, Schwindel und Meningismus kommen. In seltenen Fällen sind Hirnnervenausfälle, Hörminderung, Bewusstseinsstörungen bis hin zum Koma sowie Bewegungsstörungen möglich [8]. Ursächlich ist ein Liquorverlust, meist durch ein spinales Liquorleck [3]. Bildgebend zeigen sich mittels Magnetresonanztomographie (MRT) häufig verdickte, kontrastmittelaufnehmende Meningen, eine kaudale Hirnverlagerung, eine Erweiterung der venösen Strukturen und subdurale Hygrome. Eine spinale MRT oder kontrastmittelgestützte computertomographische (CT) oder Magnetresonanz(MR)-Myelographie, digitale Subtraktionsmyelographie oder Radioisotopenzisternographie dienen dem Nachweis und der Lokalisation des vermuteten Liquorlecks [3].

Therapeutisch steht die Bettruhe im Vordergrund. Medikamentös können Koffein, Gabapentin und Theophyllin eingesetzt werden [3]. Bei ausbleibender Besserung können ein epiduraler Blutpatch oder eine epidurale Injektion von Fibrinkleber durchgeführt werden. Zusätzlich besteht die Möglichkeit eines chirurgischen Verschlusses eines zuvor nachgewiesenen Liquorlecks [3]. Seltene Ursachen des Liquorverlusts sind Fisteln zwischen den spinalen Liquorräumen und epiduralen Venen. Insbesondere bei fehlendem Nachweis einer epiduralen Liquoransammlung sind die Fisteln eine wichtige, aber seltene Differenzialdiagnose. Die Behandlung kann chirurgisch oder auch endovaskulär mittels Embolisation der Fisteln erfolgen [1].

In der Regel kommt es innerhalb von Wochen zu einer vollständigen Remission der Beschwerden. Bei länger andauernder Symptomatik kann sich der Kopfschmerzcharakter ändern, auch die initiale Lageabhängigkeit kann einem Dauerschmerz ohne orthostatische Komponente weichen [3].

Seit 2002 wurden 49 Fälle einer frontotemporalen Demenz im Rahmen einer SIH beschrieben (Tab. 1). Der Begriff „frontotemporal brain sagging syndrome“ (FBSS) als Kombination einer frontotemporalen Demenz vom Verhaltenstyp und eines „brain sagging“ in der Bildgebung wurde 2011 durch Wicklund et al. vorgeschlagen [10].

**Table 1:** 

	Anzahl Patienten	Zeit bis Diagnosestellung	Symptomatik	Bildgebung	Therapie	Verlauf
*Ozyigit et al. *[8]	1	18 Monate	Kognitive Einschränkung Dysarthrie Choreatiforme Bewegungsstörung	„Brain sagging“	Methylprednisolon: 1000 mg i.v./Tag über 3 Tage, gefolgt von Prednisolon oral 80 mg/Tag mit wöchentlicher Reduktion um 10 mg bis auf 5 mg Tagesdosis	Klinische und bildgebende Besserung
				Meningeale Kontrastmittelaufnahme		
*Kent et al. *[6]	1	24 Monate	Wesensänderung i. S. einer frontotemporalen Demenz Okulomotorikstörung Dysdiadochokinese Dysarthrie	Transtentorielle Herniation des medialen Temporallappens	Keine	Spontane Besserung
				Meningeale Kontrastmittelaufnahme		
*Hong et al. *[5]	1	18 Monate	Wesensänderung i. S. einer frontotemporalen Demenz Kognitive Einschränkung	Kleinhirntonsillentiefstand	Prednisolon 80 mg/Tag; langsame Reduktion über 4 Monate	Klinische und bildgebende Besserung
				Meningeale Kontrastmittelaufnahme		
*Wicklund et al. *[10]	8	Median 11 Monate (6–48 Monate)	Kopfschmerz (*n* = 8) Wesensänderung (*n* = 8) Kognitive Einschränkung (*n* = 8) Tagesmüdigkeit (*n* = 8) Gangstörung (*n* = 6) Dysarthrie (*n* = 3) Bewegungsstörung (*n* = 4) Okulomotorikstörung (*n* = 3)	„Brain sagging“ mit Hirnstammkompression bei allen Patienten	Epiduraler Blutpatch (*n* = 3)	Vorübergehende Besserung bei 2 Patienten
				4/8 der Patienten mit transtentorieller Herniation des medialen Temporallappens	i.v. Methylprednisolon (*n* = 2)	1 Patient ohne Besserung, 1 Patient mit vorübergehender Besserung
				Nachweis eines Liquorlecks (*n* = 1)		
				Nachweis meningealer Divertikel (*n* = 4)	Prednisolon oral über 6 Monate	Keine klinische Besserung
					Chirurgische Intervention (*n* = 1)	Vorübergehende Besserung
*Capizzano et al. *[2]	8	Im Mittel 20 Monate	Kopfschmerz (*n* = 7) Gedächtnisstörung (*n* = 7) Tagesmüdigkeit (*n* = 7) Gangstörung (*n* = 5) Bewegungsstörung (*n* = 4) Dysarthrie und Dysphagie (*n* = 3) Vokale Tics (*n* = 2)	Kleinhirntonsillentiefstand	Epiduraler Blutpatch, epidurale NaCl-Infusion (*n* = 1)	Vorübergehende Besserung
				Verkürzter pontomamillärer Abstand		
				5/7 mit meningealer Kontrastmittelaufnahme	Spinale neurochirurgische Operation (*n* = 1)	Dauerhafte Besserung
				4/7 mit Nachweis eines spinalen Liquorlecks	Ligatur einer thorakalen Zyste (*n* = 1)	Vorübergehende Besserung
				2/7 mit Subduralhämatom	Chiari-Dekompression (*n* = 1)	Kein Effekt
					Bohrlochtrepanation (*n* = 1)	Dauerhafte Besserung
*Schievink et al. *[9]	29	Keine Angabe	Müdigkeit (*n* = 29) Kopfschmerzen (*n* = 27) Hörstörung (*n* = 21) Gangstörung (*n* = 19) Tremor (*n* = 15) Übelkeit (*n* = 19) Dysarthrie/Dysphagie (*n* = 9) Dyskinesien (*n* = 6) Inkontinenz (*n* = 4) Abduzensparese (*n* = 2) Koma (*n* = 1)	cMRT: „brain sagging“ (*n* = 29) Kleinhirntonsillentiefstand (*n* = 29) Meningeale Kontrastmittelaufnahme (*n* = 20)	Epiduraler Blutpatch (*n* = 29)	Anhaltende Besserung bei 1 Patient
				Spinale Bildgebung: meningeale Divertikel (*n* = 15)		Vorübergehende Besserung bei 24 Patienten
					Orale Glukokortikoide (*n* = 6)	Verbesserung bei 2 Patienten
					Chirurgische Behandlung (*n* = 26)	Klinische Besserung bei 21 Patienten (72 %)
*Ortega-Procayo et al. *[7]	1	16 Jahre	Kopfschmerz Gedächtnisstörung Verhaltensauffälligkeit Motorische Automatismen Dysarthrie, Dysphagie	cMRT: „brain sagging“, Kleinhirntonsillentiefstand, meningeale Kontrasmittelaufnahme	Prednisolon	Kurzfristige klinische Besserung
				CT-Myelographie: thorakale Liquor-Venen-Fistel	Operative Ligatur der Liquor-Venen-Fistel	Beschwerdefreiheit
*Gharehbagh et al. *[4]	1	1 Jahr	Kopfschmerzen Vigilanzminderung Kognitive Verschlechterung	cMRT: meningeale Kontrastmittelaufnahme, „brain sagging“	Epiduraler Blutpatch Intrathekale NaCl-Infusion	Kurzfristige klinische Besserung
				CT-Myelographie: durale Venenfistel	Operative Ligatur der Fistel	Beschwerdefreiheit

*cMRT* zerebrale Magnetresonanztomographie, *CT* Computertomographie, *NaCl* Natriumchlorid, *i.* *S.* im Sinne, *i.v.* intravenös

## Fallbericht

Eine 59-jährige Frau stellte sich in unserer Klinik aufgrund von seit 12 Monaten bestehenden, progredienten, orthostatisch auftretenden Zephalgien ohne vorangehendes erklärendes Ereignis vor. Zudem sei es in den letzten 8 Wochen zu einer deutlichen kognitiven Verschlechterung mit Orientierungsstörung, Kurzzeitgedächtnisstörung und Antriebsminderung gekommen.

Hierdurch konnte die Patientin ihren Alltag nicht mehr bewältigen, sie verbrachte den Tag überwiegend antriebsgemindert im Bett, ein adäquates Anamnesegespräch war bei Aufnahme nicht möglich, die Patientin war zeitlich und örtlich desorientiert. Eine neuropsychologische Testung ergab ausgeprägte Defizite im Bereich der Exekutivfunktionen sowie des Gedächtnisses, vereinbar mit einer frontotemporalen Störung. Weiterhin fielen eine zentrale Okulomotorikstörung mit sakkadierter Blickfolge und verlangsamten Sakkaden, eine bilaterale Hörminderung sowie eine posturale Instabilität auf.

**Figure 1:**
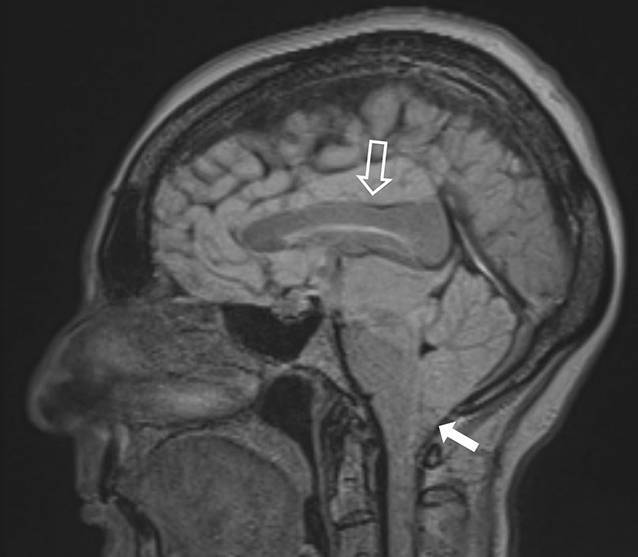

Die MRT (Abb. 1 und 2) zeigte, passend zu einer intrakraniellen Hypotension, eine Verschmälerung der äußeren Liquorräume, eine Verlagerung des Hirnstamms nach rostral, eine Absenkung des Mittelhirns, einen Tiefstand der Kleinhirntonsillen sowie eine Kontrastmittelaufnahme der Meningen.

**Figure 2:**
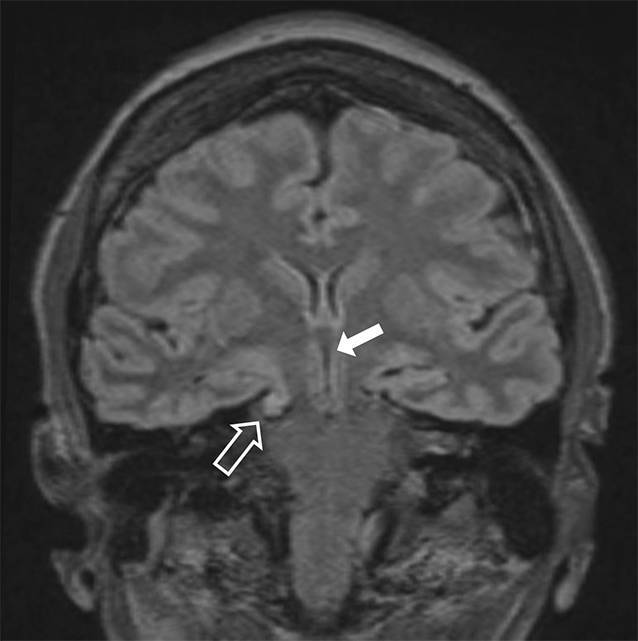

Eine native MRT der spinalen Achse konnte kein Liquorleck detektieren. Bei kaudal verlagertem Hirnstamm und aufgebrauchten infratentoriellen Liquorräumen wurde bei erhöhtem Risiko einer Herniation auf eine invasive Diagnostik mittels CT-Myelographie oder MR-Myelographie ebenso wie auf eine Liquorpunktion zur Druckmessung verzichtet.

Ein epiduraler Blutpatch wurde bei fehlendem Nachweis eines Liquorlecks, auch wenn dies einen Therapieversuch nicht ausschließt, nicht durchgeführt. Therapeutisch erfolgten strenge Bettruhe und die Gabe von Koffein (3 × 200 mg/Tag). Bei unzureichendem klinischem Ansprechen nach 7 Tagen verabreichten wir aufgrund des kasuistisch beschriebenen positiven Effektes von Glukokortikoiden (s. unten) über 6 Tage 500 mg Methylprednisolon/Tag.

Hierunter kam es innerhalb weniger Tage zu einer ausgeprägten Besserung der kognitiven Funktionen, der Gang- und Okulomotorikstörung. Die Patientin war vierfach orientiert und frei gehfähig, eine adäquate Gesprächsführung war wieder möglich. Ferner besserte sich das Hörvermögen. Nach Beendigung der Glukokortikoidtherapie kam es bei Incompliance hinsichtlich der Bettruhe innerhalb von 2 Tagen zu einer Verschlechterung der Orientierung und des Gangbildes. Nach nochmaliger 7‑tägiger Bettruhe kam es zu einer erneuten Besserung der kognitiven Funktionen und des Gangbildes. Dieser positive Trend setzte sich auch unter zunehmender Mobilisierung fort, 4 Wochen nach Beendigung der Glukokortikoidtherapie bestanden noch eine leichte Gangunsicherheit und diskrete Okulomotorikstörung. Die Patientin berichtete, dass Konzentration und Merkfähigkeit im Tagesverlauf nach längerem Stehen/Laufen nachließen, mit rascher Erholung in liegender Position. Aufgrund der anhaltenden klinischen Besserung erfolgte keine Verlaufskontrolle mittels MRT.

## Überblick über die Literatur

Es erfolgte eine systematische Literaturrecherche in Pubmed (13.12.2021; Suchterminus ((((intracranial hypotension, spontaneous[MeSH Terms])) OR (intracranial hypotension))) AND (dementia OR cognitive impairment)).

In den so identifizierten 8 publizierten Berichten wurde bei 49 Patienten eine SIH mit begleitenden Symptomen einer frontotemporalen Störung (Tab. 1) berichtet. Begleitend wurden mehrfach eine Dysarthrie, eine Bewegungsstörung [2, 7–10], eine Okulomotorikstörung [6, 9, 10] und eine Tagesmüdigkeit beschrieben [2, 9, 10].

Diagnostisch fanden sich mittels MRT die Befunde eines „brain sagging“ [7–10], mit Kleinhirntonsillentiefstand [2, 5, 7, 9] und meningealer Kontrastmittelaufnahme [2, 4–9]. Therapeutisch erfolgten ein epiduraler Blutpatch [2, 4, 9, 10], Glukokortikoide [5, 7–10] sowie die chirurgische Therapie eines Liquorlecks [2, 4, 7, 9, 10].

Alle Therapieformen konnten eine – teils nur vorübergehende – Besserung der Symptomatik bewirken, jedoch nicht bei allen Patienten. Mehrfach erfolgte im Intervall bei erneuter Zunahme der Symptomatik eine erweiterte Bildgebung zur Suche eines Liquorlecks mit anschließender chirurgischer Intervention [2, 4, 7, 9, 10]. Unter der teils kombinierten Therapie kam es unter den 49 beschriebenen Fällen bei 24 % der Patienten zu einer vorübergehenden Besserung und bei 67 % zu einer anhaltenden Besserung – bei jedoch unterschiedlich langer Nachbeobachtungszeit. Bei 9 % der Patienten konnte keine Besserung der Symptomatik erreicht werden.

## Diskussion

Wir beschreiben eine Patientin mit reversiblem frontotemporalem demenziellem Syndrom, Antriebsminderung, zentraler Okulomotorikstörung und Gangstörung bei einer SIH, welches sich unter hochdosierter Therapie mit Glukokortikoiden sowie unter konsequenter Einhaltung einer Bettruhe klinisch deutlich besserte. Auch wenn das Leitsymptom einer SIH der orthostatische Kopfschmerz ist, so ist das Spektrum der neurologischen Symptome breit. Unser Fallbericht soll deutlich machen, dass ein FBSS über viele Monate zu progredienten kognitiven Störungen führen kann und es daher in die Differenzialdiagnose einer demenziellen Entwicklung mit einbezogen werden sollte.

Als möglicher Pathomechanismus der Erkrankung wird eine direkte mechanische Einwirkung auf frontotemporale Strukturen sowie auf den Hirnstamm durch das FBSS diskutiert [5], wodurch es zu einer Beeinflussung der Verbindungen zwischen Kortex und Hirnstamm kommen könnte [10].

## Fazit für die Praxis

- Die spontane intrakranielle Hypotension ist eine Erkrankung mit vielfältigen neurologischen Symptomen.
- In der Bildgebung finden sich ein „brain sagging“, eine Kontrastmittelaufnahme der Meningen und ein Kleinhirntonsillentiefstand als Korrelate eines FBSS.
- Das FBSS ist eine wichtige Differenzialdiagnose, insbesondere zu neurodegenerativen Erkrankungen wie der frontotemporalen Demenz.
- Die therapeutischen Konsequenzen sind Bettruhe, Koffein, ggf. ein epiduraler Blutpatch, ggf. Glukokortikoide und die chirurgische Intervention bei Nachweis eines Liquorlecks.
